# Supplementary material for: A simple model suggesting economically rational sample-size choice drives irreproducibility
Source: PLoS One. 2020 Mar 11;15(3):e0229615. doi: 10.1371/journal.pone.0229615 (PMC7065751; doi:10.1371/journal.pone.0229615)
Supplement: S1 File — Code to compute the ESS (and associated parameters) based on b, d, IF. (PDF) [file pone.0229615.s001.pdf]

S1 Model Code Model code for quick reference. Code to compute the *ESS* (and associated parameters) based on b, d, IF .

```
import numpy as np
import statsmodels.stats.power as getpower

alpha = 0.05

def getESS(b,d,IF):
  """
  Calculate ESS for
  b: base rate of true hypotheses (between 0 and 1),
  d: Effect size (Cohen's d),
  IF: Income factor (# of sample pairs purchasable per publication)
  """
  SS = np.arange(4,1000,2)
  Power = np.zeros(len(SS))
  falsePR = np.zeros(len(SS))
  truePR = np.zeros(len(SS))
  totalPR = np.zeros(len(SS))
  Income = np.zeros(len(SS))
  Profit = np.zeros(len(SS))
  for i,s in enumerate(SS):
    ''' 1-sample t-test '''
    # analysis = getpower.TTestPower()
    # Power[i] = analysis.solve_power(effect_size=d, nobs=s, alpha=alpha,
    #             power=None, alternative='two-sided')
    ''' 2-sample t-test '''
    analysis = getpower.TTestIndPower()
    Power[i] = analysis.solve_power(effect_size=d, nobs1=s, ratio=1.0, alpha=alpha,
                                    power=None, alternative='two-sided')

    falsePR[i] = alpha * (1-b)
    truePR[i] = Power[i] * b
    totalPR[i] = falsePR[i] + truePR[i]
    Income[i] = totalPR[i] * IF
    Profit[i] = Income[i] - s
  ESSidx = np.argmax(Profit)
  ESS = SS[ESSidx]
  SSSidx = (np.abs(Power-0.8)).argmin()
  SSS = SS[SSSidx]
  TPR_ESS = totalPR[ESSidx]
  PPV_ESS = truePR[ESSidx]/totalPR[ESSidx]
  PPV_SSS = truePR[SSSidx]/totalPR[SSSidx]
  Power_ESS = Power[ESSidx]

  '''
  ESS = equilibrium sample size (sample size at which Profit is maximal)
  SSS = scientifically appropriate sample size (with power=80%)
  TPR_ESS = total publishable rate at ESS (describes published literature)
  PPV_ESS = positive predictive value at ESS
  Power_ESS = power at ESS
  PPV_SSS, positive predictive value at SSS
  Income = vector of income for each tested sample size
  SS = vector of tested sample sizes
  Profit = vector of profit for each tested sample size
  '''
  return ESS, SSS, TPR_ESS, PPV_ESS, Power_ESS, PPV_SSS, Income, SS, Profit
```
