# Supplementary material for: A simple model suggesting economically rational sample-size choice drives irreproducibility
Source: PLoS One. 2020 Mar 11;15(3):e0229615. doi: 10.1371/journal.pone.0229615 (PMC7065751; doi:10.1371/journal.pone.0229615)
Supplement: S2 File — Code to compute the ESSCET (and associated parameters) based on b, d, IF, Δ. (PDF) [file pone.0229615.s002.pdf]

**S2 Model Code CET** **Model code for quick reference.** Code to compute the $ESS_{CET}$ (and associated parameters) based on $b, d, IF, \Delta$ .

```
import numpy as np
import statsmodels.stats.power as getpower
from rpy2.robjects.packages import importr
import rpy2.robjects as ro

alpha = 0.05

def getESS_cet(b,d,IF):
    """
    Calculate ESS given conditional equivalence testing for
    b: baserate of true hypotheses (between 0 and 1),
    d: Effect size (Cohens d),
    IF: Income factor (# of sample pairs purchasable per publication) and
    Delta: minimally relevant effect size as a fraction of d
    """
    Delta=1

    SS = np.arange(4,1000,2)
    Power = np.zeros(len(SS))
    Power_cet = np.zeros(len(SS))
    falsePR = np.zeros(len(SS))
    truePR = np.zeros(len(SS))
    falseNR_cet = np.zeros(len(SS))
    trueNR_cet = np.zeros(len(SS))
    totalPR = np.zeros(len(SS))
    Income = np.zeros(len(SS))
    Profit = np.zeros(len(SS))
    for i,s in enumerate(SS):
        ''' 1-sample t-test '''
        # analysis = getpower.TTestPower()
        # Power[i] = analysis.solve_power(effect_size=d, nobs=s, alpha=alpha,
        #                power=None, alternative='two-sided')
        ''' 2-sample t-test '''
        analysis = getpower.TTestIndPower()
        Power[i] = analysis.solve_power(effect_size=d, nobs1=s, ratio=1.0, alpha=alpha,


        falsePR[i] = alpha * (1-b)
        truePR[i] = Power[i] * b

        ''' under cet studies not finding a significant positive result
        are tested for significant negative results, where the latter are
        determined by two one-sided t-tests (TOST, Campbell and Gustafson,
        2018). TOST power calculation from R TOSTER package (Lakens 2017)'''

        R_command = 'TOSTER::powerTOSTtwo(alpha=0.05, N='+str(s)+', low_eqbound_d='+
                                str(-Delta*d)+', high_eqbound_d='+str(Delta*d)+')'
        Power_cet[i] = ro.r(R_command)[0]
        falseNR_cet[i] = alpha * b * (1-Power[i])
        trueNR_cet[i] = Power_cet[i] * (1-b) * (1-alpha)
        '''the correction factors (1-Power[i]) and (1-alpha) above account
        for the fact that positive results will not be subjected to the
        equivalence test.'''

        ''' here (with CET) total 'publishable' rate incorporates
        significant alternative hypotheses as well as
        significant null hypotheses '''
        totalPR[i] = falsePR[i] + truePR[i] + falseNR_cet[i] + trueNR_cet[i]
        Income[i] = totalPR[i] * IF
        Profit[i] = Income[i] - s
    ESSidx = np.argmax(Profit)
    ESS = SS[ESSidx]
    SSSidx = (np.abs(Power-0.8)).argmin()
    SSS = SS[SSSidx]
```

```
TPR_ESS = totalPR[ESSidx]
PPV_ESS =  (truePR[ESSidx]+trueNR_cet[ESSidx])/totalPR[ESSidx]
P_cet =  Power_cet[ESSidx]
Power_ESS = Power[ESSidx]

'''
ESS = equilibrium sample size (sample size at which Profit is maximal)
SSS = scientifically appropriate sample size (with power=80%)
TPR_ESS = total publishable rate at ESS (describes published literature)
PPV_ESS = positive predictive value at ESS
Power_ESS = power at ESS
P_cet, power of CET at ESS
Income = vector of income for each tested sample size
SS = vector of tested sample sizes
Profit = vector of profit for each tested sample size
'''
return ESS, SSS, TPR_ESS, PPV_ESS, Power_ESS, P_cet, Income, SS, Profit
```
